# GAPscreener: An automatic tool for screening human genetic association literature in PubMed using the support vector machine technique

**DOI:** 10.1186/1471-2105-9-205

**Published:** 2008-04-22

**Authors:** Wei Yu, Melinda Clyne, Siobhan M Dolan, Ajay Yesupriya, Anja Wulf, Tiebin Liu, Muin J Khoury, Marta Gwinn

**Affiliations:** 1 National Office of Public Health Genomics, Coordinating Center for Health Promotion, Centers for Disease Control and Prevention, Atlanta, GA, USA; 2 Albert Einstein College of Medicine/Montefiore Medical Center, Bronx, NY, USA

## Abstract

**Background:**

Synthesis of data from published human genetic association studies is a critical step in the translation of human genome discoveries into health applications. Although genetic association studies account for a substantial proportion of the abstracts in PubMed, identifying them with standard queries is not always accurate or efficient. Further automating the literature-screening process can reduce the burden of a labor-intensive and time-consuming traditional literature search. The Support Vector Machine (SVM), a well-established machine learning technique, has been successful in classifying text, including biomedical literature. The GAPscreener, a free SVM-based software tool, can be used to assist in screening PubMed abstracts for human genetic association studies.

**Results:**

The data source for this research was the HuGE Navigator, formerly known as the HuGE Pub Lit database. Weighted SVM feature selection based on a keyword list obtained by the two-way z score method demonstrated the best screening performance, achieving 97.5% recall, 98.3% specificity and 31.9% precision in performance testing. Compared with the traditional screening process based on a complex PubMed query, the SVM tool reduced by about 90% the number of abstracts requiring individual review by the database curator. The tool also ascertained 47 articles that were missed by the traditional literature screening process during the 4-week test period. We examined the literature on genetic associations with preterm birth as an example. Compared with the traditional, manual process, the GAPscreener both reduced effort and improved accuracy.

**Conclusion:**

GAPscreener is the first free SVM-based application available for screening the human genetic association literature in PubMed with high recall and specificity. The user-friendly graphical user interface makes this a practical, stand-alone application. The software can be downloaded at no charge.

## Background

The peer-reviewed scientific literature is a major source of information for developing research hypotheses and creating new knowledge through synthesis of research findings [1]. The information explosion in biomedical science has created a huge challenge for researchers, who want to obtain useful information promptly and efficiently. Human genetic association studies epitomize this challenge because they have proliferated rapidly since completion of the Human Genome Project [2]. Systematic review and meta-analysis have become important approaches for evaluating the robustness of such associations across different study platforms and populations [3]. A key factor in the quality of a systematic review is complete capture of the relevant studies [4]. Many databases that deposit genetic association information, including citations from PubMed, have been built and curated [5-7]. PubMed [8] is the largest publicly accessible biomedical literature database and is the main source for such activities. However, because of its large size and the complex syntax required for query formation, it is fairly difficult to comprehensively and effectively search PubMed for genetic association studies. The necessarily labor-intensive screening and curation process makes the maintenance of such databases extremely challenging.

Automatic literature classification is becoming increasingly attractive and has already demonstrated some successes in the biomedical literature [9-12]. The support vector machine (SVM) method [13] is a powerful machine learning technique that has been used to solve classification problems [14-18]. An earlier report described a potential application of SVM methods to classify literature on human genome epidemiology [10]. In this paper, we report a novel method for feature selection and show that using it to train the SVM model significantly improved its ability to classify reports of human genetic association studies. We implemented the method as a Java-based application named GAPscreener (**G**enetic **A**ssociation **P**ublication screener) that can be freely downloaded [19].

## Implementation

### SVM Model Generation

#### Data sources

To generate the training dataset for the SVM experiment, we used 10,000 randomly selected abstracts from articles published between 2001 and 2006 in PubMed as a background dataset. The positive dataset consisted of 10,000 randomly selected gene-disease association articles from the HuGE Navigator [5] (formerly known as the HuGE Pub Lit database [6]), a continuously updated database of studies relevant to human genome epidemiology sponsored by the National Office of Public Health Genomics. Inclusion and exclusion criteria for positive dataset from the HuGE Pub Lit database has been reported [6].

#### PubMed abstract text retrieval

We developed a PubMed text extraction tool using the NCBI E-utility [20] to retrieve text content based on PubMed identification numbers (PMIDs). The text used for processing consisted of the title and the abstract, or the title alone if the abstract was not available. The text data were stored in a data structure for processing.

#### Text processing and extraction of keywords

The abstract and title of each article were then processed with the text-processing tool we developed. A stemming technique was used to deal with morphologic word changes, for example, polymorph(isms) and polymorph(ic) were considered the same word. A stop word list was generated for some common English words, such as pronouns and articles, to reduce the number of words extracted.

#### Significant keyword generation

We selected keywords by identifying statistically significant differences between the probability of their occurrence in the text (title and abstract) of human genetic association articles, compared with their frequency in all other articles. The sample sizes of both groups were large enough that the distribution of differences in probabilities was approximated by a normal distribution. Thus words with a z score greater than 1.96 or less than – 1.96 (significance level of α = .05) were chosen as feature keywords.

The statistical formula [21] used for calculating the z score is given by:

$$\begin{array}{c} \text{Z}=\frac{{\text{p}}_{1}-{\text{p}}_{2}}{\sqrt{\text{pq}(\frac{1}{{\text{n}}_{1}}+\frac{1}{{\text{n}}_{2}})}}\text{ if }({\text{n}}_{1}\text{pq}>5\&({\text{n}}_{2}\text{pq}>5)\\ \begin{array}{cc} \text{p}=\frac{{\text{n}}_{1}{\text{p}}_{1}+{\text{n}}_{2}{\text{p}}_{2}}{{\text{n}}_{1}+{\text{n}}_{2}} & \text{q}=1-\text{p} \end{array} \end{array}$$

where:

p_1 _= probability of occurrence of word in genetic association abstracts.

p_2 _= probability of occurrence of word in non-genetic association abstracts.

n_1 _= total occurrences of word in genetic association abstracts.

n_2 _= total occurrences of word in non-genetic association abstracts.

#### Generating SVM input data

The statistically significant keywords are called feature keywords and were used to construct the SVM features. Each feature keyword was weighted according to its z score, normalized to values from -1 to +1. For the training and testing data sets, the script generated the SVM input based on sparse format [22]. The presence of each keyword was represented by its position on the feature keyword list, followed by a colon and the normalized z score; the absence of keywords was ignored and each feature was separated by a space, for example, 1:0.003589 30:- 0.81189. In the training data set, the first column of the input data was set to the known outcome, i.e., 1 for positive, -1 for negative. In the test set, the first column of the input dataset was set to 0.

Two sets of significant keywords were generated. One set contained those with positive z scores above the threshold (1.96) (called one-way weighted scheme); the other contained key words with both positive (greater than 1.96) and negative z scores (less than -1.96) (called two-way weighted scheme).

#### SVM model training

We used LibSVM [22], a freely available SVM software library, to train the SVM model. The accompanying utility, grid.py, was used to find optimum parameters for penalty parameter C and gamma in the radial basis function (RBF) kernel. The RBF kernel was chosen based on its potential in terms of performance [23].

### Stand-alone Application Implementation

GAPscreener is a stand-alone application built with the Java programming language. Java Swing [24] components were used to build the graphical user interface (GUI). The application incorporates open-source LibSVM Java codes for prediction, employing the SVM model we trained. Java-based Web services in the NCBI E-utility were used to query and retrieve PubMed records. EzInstall [25], a freeware application, was used to package the application with a Java Runtime Environment (JRE), for automatic, self-contained installation.

### Performance Evaluation

#### General performance evaluation

To evaluate the performance of the screening tool, we used a series of new test data (not included in the training set). The first test data set (92253 negatives, 773 positives) consisted of selections from PubMed during five consecutive weeks (February 22, 2007 to March 22, 2007) according to the routine, traditional screening process used to build the HuGE Navigator [5]. Positive or negative status assigned by the routine process was considered the gold standard. We used this data set to evaluate two keyword weighting schemes. A second data set (68255 negatives, 597 positives), selected from PubMed during four subsequent weeks (April 5, 2007 to April 26, 2007), was used to evaluate false-positive results generated by the GAPscreener using the selected weighting scheme.

Recall, specificity and precision were calculated from the test data to evaluate the performance of the application. The formulas for calculating these parameters are as follows:

Re⁡call=TPTP+FNPr⁡ecison=TPTP+FPSpecificity=TNTN+FP

where TP, TN, FP and FN represent the number of true positive, true negative, false positive and false negative results, respectively.

To compare the results of classification by the SVM tool with the gold standard, we used logistic regression (SAS Version 9.13, SAS Institute, Cary, NC). We produced separate logistic regression models for results of the one-way and two-way SVM schemes during the 5-week experiment (February 22, 2007 to March 28, 2007). Results from each model were used to generate receiver-operating characteristics (ROC) and calculate the area under the curve (AUC) with 95% confidence intervals. The AUC of ROC curves for the two models were compared using nonparametric methods [26,27].

#### Domain-specific performance evaluation

A list of articles compiled independently by domain experts was used as the gold standard to evaluate the predictive accuracy of the application. A network of eight experts in the analysis of genetic associations with preterm birth performed a comprehensive literature search to build a knowledge base for systematic review and meta-analysis. The search was limited to articles published from January 1, 1990, to April 12, 2007. Complex queries compiled by a librarian were used to query PubMed and EMBASE [28]. The complex queries consisted of sophisticated PubMed and EMBASE syntax filling more than four single-spaced pages. The results were manually reviewed by the domain experts.

For comparison, we used the GAPscreener to screen all PubMed abstracts published during the same period of time in a two-step process. First, we compiled a broad PubMed query based on common terms related to preterm birth. The 42,585 PubMed abstracts returned by this query were then classified by the SVM tool.

Query: Prematurity OR infant, premature OR infant, low birth weight OR labor, premature OR preterm labour OR premature birth OR preterm birth OR preterm infant OR preterm premature rupture OR preterm pregnancy outcome OR preterm delivery OR adverse outcomes of pregnancy OR obstetric labor, premature.

## Results

### SVM feature selection

We generated a list of significant keywords using the z score method, based on comparing their relative frequencies in 10,000 general PubMed abstracts and 10,000 gene disease-associated abstracts included in the HuGE Pub Lit database. The one-way and two-way weighted schemes generated lists of 1,301 and 4,589 keywords, respectively. Normalized z scores between 1 and -1 were used as weighting parameters for each keyword.

The two-way weighted scheme (using keywords with positive and negative z scores) performed better than the one-way scheme in terms of recall, specificity and precision (Table 1). The AUC for the two-way scheme was significantly larger than for the one-way scheme (p < 0.0001).

**Table 1:** Performance test results comparing SVM results with known classification in test set (data selected from PubMed during five consecutive weeks from Feb 22, 2007 to March 28, 2007)

	**Test Parameters**	**22-Feb-07**	**1-Mar-07**	**8-Mar-07**	**15-Mar-07**	**22-Mar-07**	**ROC area (95% CI)**	***p *value**
One Way	Recall	0.946	0.968	0.951	0.965	0.951	0.967	
	Precision	0.345	0.297	0.265	0.298	0.265	(0.958–0.975)	
	Specificity	0.981	0.981	0.980	0.981	0.980		
								< 0.0001
Two Way	Recall	0.946	0.992	0.967	0.977	0.993	0.982	
	Precision	0.345	0.311	0.291	0.323	0.336	(0.976 – 0.987)	
	Specificity	0.981	0.982	0.982	0.983	0.984		

One-way: key words with z scores greater than 1.96 were selected as featured key words.

Two-way: key words with z scores greater than 1.96 or less than -1.96 were selected as featured key words.

AUC: area under the curve.

CI: confident interval

### Using the SVM tool for HuGE Pub Lit database screening and curation

The routine screening process used to perform weekly updates of the HuGE Pub Lit database was based on a complex query that combined Medical Subject Headings (MeSH) terms and selected text words, followed by a labor-intensive, time-consuming manual review by a single curator (MC) [5]. Because a previous evaluation had concluded that the recall of this process was about 80% [5], we re-evaluated the SVM false positives and found that the SVM was able to pick up 47 positive articles missed by the traditional curation process during the 4-week evaluation period; however, 14 positive abstracts were missed by the SVM (Table 2).

**Table 2:** Results of the SVM method and previous method in screening PubMed for the HuGE Pub Lit database.

	**05-Apr-07**	**12-Apr-07**	**19-Apr-07**	**26-Apr-07**
Number of positive abstracts missed by the previous method*	22	17	5	3
**Number of positive abstracts missed by SVM**	5	4	1	4
**Number of positive abstracts picked up by both methods**	179	159	131	114
**Number of total positive abstracts**	206	180	137	121

* True positives re-evaluated by the curator.

The number of abstracts returned by the query is a crucial factor in determining the burden of curating the HuGE Navigator database. The ever-increasing number of genetic association studies – combined with curator fatigue – may also influence the quality of the database. Our 4-week experiment showed that using the GAPscreener reduced the number of abstracts requiring manual review approximately 8-fold (Table 3).

**Table 3:** Numbers of PubMed abstracts requiring manual review after screening by SVM method and previous method*.

	**05-Apr-07**	**12-Apr-07**	**19-Apr-07**	**26-Apr-07**	**Total**
**The SVM tool**	521	397	458	400	1776
**The previous method**	4010	3013	3789	3382	14194

Note: the number for the SVM tool was generated based on Entrez date; the number for the previous method was generated based on MeSH date.

*: Previous method: the screening method described in the reference 5.

### Screening PubMed for genetic associations with preterm birth

**Figure 1:**
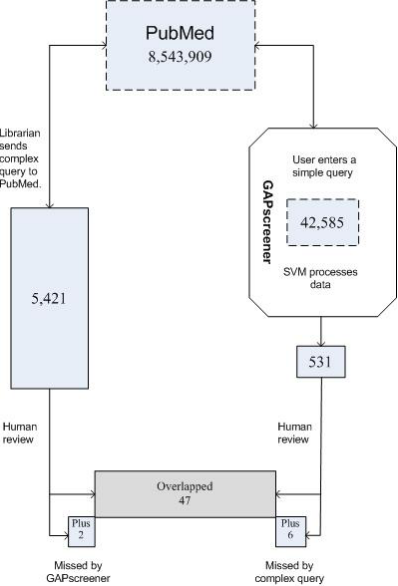
**Results of traditional search method compared with use of GAPscreener (preterm birth example)**. Both methods searched all PubMed abstracts entered from January 1, 1990 through April 12, 2007. Numbers indicate the number of PubMed abstracts processed at each stage.

We built this application not only for general screening of the PubMed literature on genetic associations but also as a tool that could be customized for searching genetic association literature in any specific domain. We used preterm birth as an example to evaluate the application's performance in this setting. An independent screening process performed by domain experts first identified 5,421 articles in PubMed and EMBASE by complex PubMed and EMBASE queries. After reviewing each abstract manually, 49 articles were included in the knowledge base. All 49 articles were recorded in the PubMed database. In a parallel process, the GAPscreener was used to perform the initial screening automatically with the preterm birth specific query (see Method), identifying 531 articles. Of these, 47 (96%) overlapped with the set of 49 articles identified by the domain experts. The GAPscreener missed two articles found by the traditional process but picked up six additional articles that the traditional process had missed (Figure 1).

### Implementation of the user-friendly application

**Figure 2:**
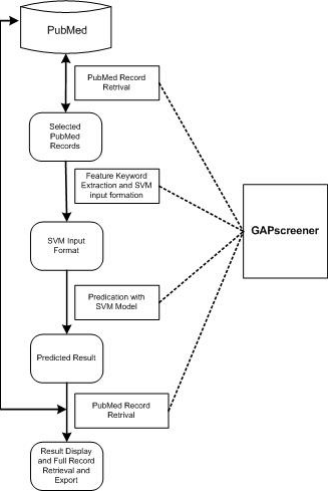
Data flow scheme in GAPscreener's screening process.

**Figure 3:**
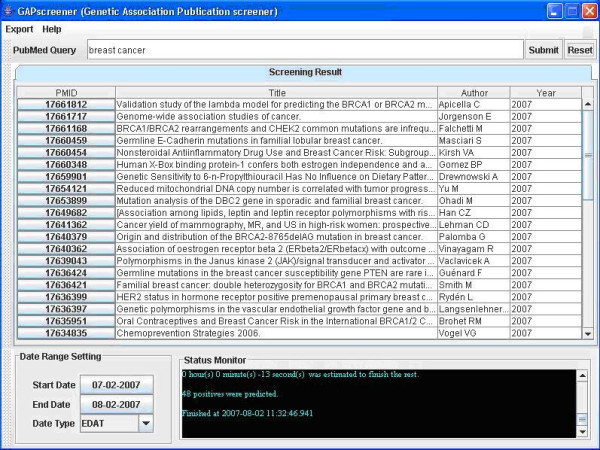
Graphical user interface (GUI) of GAPscreener.

The GAPscreener includes all components in the screening process: PubMed record retrieval from NCBI, text content processing for keyword extraction, SVM input data formatting, and SVM output display and record export (Figure 2). A graphical user interface (GUI) provides a user-friendly environment (Figure 3). The application can be freely downloaded and its self-installation capacity makes the process fairly easy.

## Discussion

The number of published genetic associations has exploded during the past decade [6]. Finding these associations in major online databases like PubMed is critical for establishing the knowledge base on genetic factors in specific diseases [7]. Automated tools are needed to help scientists cope with the information overload. For 6 years, the HuGE Pub Lit database has continuously collected PubMed literature related to human genome epidemiology, providing a great opportunity to test machine learning techniques for automating the screening process [28]. Compared with the existing, traditional screening process, the GAPscreener dramatically reduced the burden of manual review and substantially improved screening recall, from 80% to 97.5%.

Feature selection is an important element of the support vector machine technique. Our weighted z score method performed better than a previously reported method based on the Term Frequency × Inverse Document Frequency (TFIDF) weighting scheme [10]. Representing statistical information for each keyword as a normalized z score (value between 1 and -1) performed better than the binary representation [10].

As we demonstrated in the example of preterm birth, a potentially important application of the GAPscreener is identifying genetic association literature in a specific domain (e.g., disease, gene, or pathway). This could be very useful to disease-specific networks or consortia, such as those that have banded together in a global HuGENet collaboration [30]. The GAPscreener takes advantage of PubMed search capacity to narrow down the returned abstracts to a specific topic before applying the SVM technique.

The GAPscreener could become a routine screening tool for researchers and database curators for maintaining a local reference database. The tool can be downloaded at no charge and source code is available upon request. It is a freeware search tool that can assist researchers with systematic reviews by identifying genetic association literature in PubMed in a user-friendly and sensitive way. To our knowledge, it is the first free application that uses SVM techniques to classify published literature related to human genetic association studies. Certainly, a similar approach could be used to classify literature in other biomedical fields.

Although the GAPscreener demonstrated high recall and specificity, it has many aspects that could be improved. For example, the two-way weighted z score scheme based on a threshold of ± 1.96 generated 4,589 keywords. The number of featured keywords influences the processing speed, which in this example averaged about 0.02 second per abstract. We are planning to experiment with shorter featured keyword lists to improve processing time without sacrificing recall.

The keyword approach is only one of many ways to transform text into a feature vector. Use of controlled vocabularies can make "keywords" more meaningful and condense the list by reducing synonyms for a particular concept to a single term. The Unified Medical Language System (UMLS) sponsored by the National Library of Medicine provides a central repository for standard controlled vocabularies in the biomedical fields [31]. MetaMap Transfer (MMTx) is a tool that maps free text to concepts in the UMLS Metathesaurus [32]. UMLS terms could be used during the selection of featured keywords.

## Conclusion

GAPscreener is the first free SVM-based application available for screening the human genetic association literature in PubMed. It uses a novel SVM weighted-feature selection scheme. A performance evaluation demonstrated high recall and specificity. The user-friendly graphical user interface makes this a practical, stand-alone application.

## Competing interests

The authors declare that they have no competing interests.

## Availability and requirements

Project home page:



Operating systems: Windows

Programming language: Java

Software packages: J2EE 1.4.

License: GNU General Public License. This license allows the source code to be redistributed and/or modified under the terms of the GNU General Public License as published by the Free Software Foundation. The source code for the application is available at no charge.

Any restrictions to use by non-academics: None

## Authors' contributions

WY designed and implemented the infrastructure, wrote the source codes, and drafted the manuscript. MC was involved in the data curation and evaluation tests. SD was involved in the test data preparation and evaluation. AY was involved in the data analysis and helped in manuscript preparation. AW participated in design of the system evaluation, data collection and analysis. TL performed the statistical design and data analysis. MG provided advice on the project and revised the draft manuscript and led the project. MJK oversaw the project and revised the draft manuscript. All authors read and approved the final document.
